# Pilot Ecological Momentary Assessment Study of Subjective and Contextual Factors Surrounding E-Cigarette and Combustible Tobacco Product Use among Young Adults

**DOI:** 10.3390/ijerph182111005

**Published:** 2021-10-20

**Authors:** Deepa R. Camenga, Angela M. Haeny, Suchitra Krishnan-Sarin, Stephanie S. O’Malley, Krysten W. Bold

**Affiliations:** 1 Department of Emergency Medicine, Yale School of Medicine, New Haven, CT 06519, USA; 2 Department of Psychiatry, Yale School of Medicine, New Haven, CT 06519, USA; angela.haeny@yale.edu (A.M.H.); suchitra.krishnan-sarin@yale.edu (S.K.-S.); stephanie.omalley@yale.edu (S.S.O.); krysten.bold@yale.edu (K.W.B.)

**Keywords:** tobacco, young adult, e-cigarette

## Abstract

Background: Dual use of e-cigarettes and combustible tobacco products is common in young adults. We aimed to explore how ratings of subjective and contextual factors differed between discrete episodes of e-cigarette use vs. combustible tobacco product smoking among a sample of young adults. Methods: Young adults (N = 29, ages 18–30) who used e-cigarettes and ≥1 combustible tobacco product at least once weekly completed a 1-week smartphone-based ecological momentary assessment (EMA). Twice daily random prompts assessed past-15-min use of tobacco products, ratings of subjective factors (e.g., negative affect, craving), and contextual factors related to activity, location, and companionship. A multivariable GEE model assessed whether subjective or contextual factors were associated with e-cigarette vs. combustible tobacco product episodes. Results: 184 tobacco use episodes were reported (39.7% e-cigarette, 60.3% combustible tobacco product). High baseline cigarette dependence, as measured by the Fagerström Test for Cigarette Dependence, was associated with lower odds of e-cigarette vs. combustible tobacco product episodes (aOR 0.01, 95% CI (0.002–0.08); *p* < 0.001). Neither between- or within-subjects negative affect or craving scores were associated with e-cigarette use. Activities of eating/drinking (aOR 0.20, 95% CI (0.08–0.49); *p* = 0.001) and being in the companionship of a person who smoked cigarettes (aOR 0.13, 95% CI (0.04–0.43); *p* = 0.001) were associated with lower odds of e-cigarette vs. combustible tobacco product use episodes. However, traveling (aOR 12.02, 95% CI (3.77–38.26); *p* ≤ 0.001) and being in a public space (aOR 2.76, 95% CI (1.10–6.96); *p* = 0.03) were associated with higher odds of e-cigarette than combustible tobacco product use episodes. Conclusions: This pilot data suggests that unique contextual factors may be associated with e-cigarette use, compared to combustible tobacco smoking in a sample of young adults who use both e-cigarettes and combustible tobacco products. Future research with larger samples is needed to better characterize varying contexts and cues for tobacco use among young adults who are dual users.

## 1. Introduction

Use of both combustible cigarettes and e-cigarettes (“dual use”) is the most common poly-tobacco use pattern among U.S. young adults [1]. From a public health perspective, dual use has been argued to have the potential to reduce the overall burden of tobacco-related disease if it is part of a trajectory of combustible tobacco product cessation. A 2021 Cochrane Systematic Review reported “moderate certainty” that use of nicotine-containing e-cigarettes increases combustible cigarette cessation rates as compared to nicotine replacement therapy [2]. Further, a longitudinal analysis of data from the Population Assessment of Tobacco and Health (PATH) Study, an ongoing U.S. nationally representative prospective cohort study on tobacco product usage and health, found a tobacco cessation rate (inclusive of all tobacco products) of 7% at 12 months among adults who use e-cigarettes and cigarettes [3]. However, it is also possible that dual use could pose a significant public health risk if it prolongs and sustains nicotine addiction or decreases the likelihood of quitting all tobacco products. Given the current state of evidence, a better understanding of dual use is needed to inform the overall public health impact of e-cigarettes. 

As a first step in understanding how to best assist young adults in achieving tobacco cessation, it is necessary to better understand naturalistic patterns of dual tobacco product use. Previous research has demonstrated that distinct subjective and contextual factors trigger discrete episodes of naturalistic tobacco use. For example, negative affect is often reported as a principle motive for cigarette smoking [4], and is a potent stimulus for lapsing [5]. Cigarette smoking is also correlated with social cues (being around smokers), certain activities (i.e., eating, alcohol use), and certain locations (being at home vs. places with smoking restrictions) [6,7]. Because e-cigarettes can sometimes be used in traditionally smoke-free venues and currently have higher social acceptability than traditional cigarettes, it is plausible that young adults may use e-cigarettes in different socio-environmental contexts than cigarettes.

Ecological momentary assessment (EMA) provides an important method to study the subjective and contextual factors associated with e-cigarette and combustible tobacco product use, as it collects data in real-world settings, and uses multiple assessments over time to characterize behavior and experiences that are less prone to recall bias [8,9]. Although researchers have used EMA to extensively describe naturalistic cigarette use, to our knowledge, only three studies have examined dual e-cigarette and cigarette use in young adults [7,10,11]. These studies have begun to examine several important issues related to dual use, such as the feasibility of using EMA for valid e-cigarette measurement [10], the correlation between same-day e-cigarette use and cigarette smoking frequency [7], and whether alcohol/drug use or being alone or with others predict in the moment cigarette, e-cigarette, or other tobacco product use [11]. However, to date, no EMA studies have provided a detailed assessment of the contextual factors that surround in-the-moment naturalistic use of e-cigarettes among young adults who are dual users in real-world environments.

This pilot EMA study aims to explore how ratings of subjective (affect, craving) and contextual (location, activity, and companionship) factors differ between discrete episodes of e-cigarette use and combustible tobacco product smoking among a sample of young adults. We focus this study on young adults who use both e-cigarettes and combustible tobacco products due to the high prevalence of this dual use pattern in this population [1].

## 2. Materials and Methods

This pilot study used smartphone-based ecological momentary assessment to gather real-time data of e-cigarette and combustible tobacco product behaviors among non-treatment seeking young adults during a 1-week naturalistic dual use period. After completing a baseline in-person visit to confirm eligibility criteria and complete baseline measures, participants responded to twice daily random prompts for 1 week assessing in-the-moment use of e-cigs and combustible tobacco products, subjective factors (ratings of affect and craving), and contextual factors (location, activity, companionship) associated with each tobacco use episode. After the 1-week EMA period, the participants completed one in-person follow up visit to review their EMA adherence. Participants were remunerated $15 for each in person visit (baseline and follow-up). To encourage EMA participation, they also could receive an additional $50 for submitting at least 1 EMA response and an additional $14 if they completed at least 85% of the EMA prompts.

Study procedures were approved by the Yale Human Investigations Committee (the local Institutional Review Board) and participants provided written consent (protocol code 1612018726; approved 18 January 2017).

### 2.1. Participants and Settings

Participants were recruited between February and October 2018 through flyers, targeted advertisements on Facebook, Instagram, Craigslist, public boards in local colleges, and through the university research volunteer database. These recruitment portals directed young adults to complete an online screening form, wherein they completed a confidential questionnaire to assess eligibility. Young adults were eligible for study participation if they were between 18 through 29 years of age; used at least one type of combustible tobacco product (cigarettes, cigars, cigarillos, hookah, roll your own cigarettes) on at least 1 day during the past 7 days; used e-cigarettes on at least one day during the past 7 days; had access to an e-cigarette for personal use; were fluent in English; had a functioning cell phone for personal use with wireless, camera, and application capability (via Apple or Android platform); had access to wireless networks at least once daily; and had self-reported good health. Exclusion criteria included current enrollment in any substance use or smoking cessation program/research study; interest in using smoking cessation pharmacotherapy during the study; use of any psychoactive medications; and pregnancy or breastfeeding.

Participants who met eligibility criteria were scheduled for an in-person assessment with the study research assistant. During the in-person assessment, participants provided written consent for study participation. They were re-screened for eligibility in-person, and women provided a urine sample to confirm pregnancy status. Participants completed a battery of baseline questionnaires (See Section 2.3). The research assistant then guided the participant to download the EMA software on their personal cell phone and provided standardized training on study procedures and use of the smartphone EMA data collection tool. Participants were instructed to continue smoking combustible tobacco products and using e-cigarettes ad libitum without changing their tobacco use frequency/pattern during the 1-week study period. They were also instructed to use their own combustible tobacco products, e-cigarettes, and e-liquids during the study.

### 2.2. EMA Procedures

EMA data was collected via the MetricWire iOS and Android mobile applications (“apps”) for EMA data collection [12]. Data generated from the app was stored on a HIPAA and 21 CFR Part 11 compliant servers and encrypted during transit. Study investigators accessed EMA data through an encrypted web-based application.

For 7 days, participants were prompted to complete 3 surveys a day via the EMA application. Two EMA surveys were daily random prompts and the third was a daily nighttime assessment wherein participants reported their past-24-h combustible tobacco product and e-cigarette consumption. The daily random prompts were sent out within two random four-hour time blocks during the participant’s waking hours and the daily nighttime diary was sent at 8 pm every night. Participants had 30 min to respond to random prompts before the response portal closed; in this case, data was considered lost. Each random prompt was date- and time-stamped and recorded whether the assessment was completed or missed during the 30-min data collection period.

### 2.3. Measures

#### 2.3.1. Baseline Visit

At the baseline study visit, participants completed surveys to collect demographic information (age, sex, race, ethnicity, education, health insurance status (as a proxy of socioeconomic status)). Trained research staff also administered a 28-day timeline followback (TLFB) interview, a validated method to obtain self-report estimates of cigarette smoking as well as other substance use [13,14,15]. In this study, the TLFB assessed combustible tobacco product use (cigarettes, cigars cigarillos, hookah, roll your own cigarettes, other combustible tobacco products) and e-cigarette use (number of times used e-cigarette/day, number of puffs/day, refill of e-liquids (yes/no)) in the past 28 days. Participants also completed the Fagerström Test for Cigarette Dependence (FTCD) [16]. They were instructed to bring their preferred e-cigarette to the baseline study visit and refer to it while completing survey measures assessing e-cigarette characteristics including device type (disposable, cartridge, tank, mod/build your own). 

#### 2.3.2. EMA Measures: The Random EMAs Collected the Following Items

Tobacco Product Use: The random surveys first assessed whether the participant had used an e-cigarette only, combustible tobacco product only, or both products in the last 15 min (yes/no). Due to the small sample size of dual use episodes (defined as use of both e-cigarette and combustible tobacco products in the past 15 min; *n =* 22 of 184 total episodes), episode types were classified as e-cigarette use only or combustible tobacco product smoking (inclusive of both combustible tobacco product only and dual use episodes) for the current analysis.

- Subjective Factors: Affect was measured via the International Positive and Negative Affect Schedule Short Form’s 5-item measure of negative affect [17]. Participants rated their emotions on a 5-point scale (1 = very slightly/not at all; 2 = A little; 3 = Moderately; 4 = Quite a bit; 5 = Extremely) in response to the following questions: “During the past 15 min how much have you felt: (upset, hostile, ashamed, nervous, afraid). The negative affect score was derived from the summed responses from the five items (range 5–25). Craving for combustible tobacco and e-cigarettes was measured individually with two questions “In the past 15 min, how strong has your urge been to (smoke/vape)?” (0 = no urges; 1 = slight; 2 = moderate; 3 = strong; 4 = very strong; 5 = extremely strong) [18]. For dual use episodes, the average of the ratings for the combustible tobacco and e-cigarette questions was used to assess tobacco craving.
- Contextual Factors: Participants were asked “During this smoking/vaping episode, what were you doing?” (eating/drinking a non-alcoholic beverage, drinking alcohol, working/reading/studying, traveling, socializing, other) to measure their current activity. Eating/drinking a non-alcoholic beverage and drinking alcohol were combined into one category “eating/drinking”.
- Location: Was measured with the question “During this smoking/vaping episode, where were you?” (home, work/school, vehicle, bar/restaurant/store, other). Work/school, bar/restaurant/store, or other were combined into one category of public space (yes/no).
- Companionship: Was measured with the question “During this smoking/vaping episode, who were you around?” (smoker, person who is vaping, non-smoker, alone). Responses were recoded to a dichotomous variable of tobacco user, inclusive of being with a smoker or person who is vaping (yes/no).

## 3. Data Analysis

The data were analyzed in SAS 9.4. (SAS Institute, Cary, NC, USA) using generalized estimating equations (GEE) with a binomial distribution. Tobacco use episodes were categorized as 1 = combustible tobacco product (inclusive of both combustible tobacco product only and dual use episodes) and 2 = e-cigarette only episodes. Combustible tobacco product episodes were the reference group in all models. Separate bivariate GEE models assessed relationships between the outcomes (e-cigarette only vs. combustible tobacco product episodes), and predictors which included age, sex, education status (finished 1 or more years of college vs. high school or less), insurance status (public/no insurance (yes/no)), baseline FTCD score, e-cigarette device type (cartridge (yes/no), tank (yes/no), other device (inclusive of disposables, mods, and other devices (yes/no)) and each of the subjective and contextual factors. We used a purposeful covariate selection technique wherein variables that met the significance level of *p* < 0.20 in bivariate analyses were included in the multivariable regression model [19]. For both bivariate and multivariable models, craving and negative affect were centered on the mean and entered in the model to examine within and between-person effects. Categorical predictors were entered in the model as binary variables. Multivariable models were adjusted for timing of the assessment (weekend (Friday, Saturday, or Sunday), time of day (am vs. pm), and day of study (1 to 7)) and the significance level was 0.05.

We also performed a sensitivity analysis to explore whether the findings changed when we excluded dual use episodes (*n =* 22 of 184 total episodes). The sensitivity analysis compared e-cigarette only episodes to combustible tobacco product-only episodes (*n =* 162 total episodes) using the same methods as the primary data analysis.

## 4. Results

The sample included 29 young adult participants (mean age 22.9 years old [SD = 3.4], 48% women; See Table 1). The majority (62%) reported high school as their highest level of education completed, and the majority (59%) had public health insurance. The most common e-cigarette device types used were cartridges and tanks. Results from the 28-day TLFB showed that participants reported a mean of 4.9 e-cigarette use episodes per day, with a median of 0.07 refills per day. All participants used cigarettes, and 27% used additional tobacco products of hookah and/or cigars. In terms of cigarette use, 35% reported smoking 1 to 5 cigarettes/day and 31% reported non-daily cigarette smoking. 

Participants completed a total of *n* = 305 random EMA prompts (of which *n =* 184 were tobacco use episodes (60.3% of total)). The median EMA adherence rate was 69%, defined as completing at least 85% of the EMA prompts. The average time to complete a daily random prompt was 1.2 min (range 0.5–3.5 min). The GEE models examined data from 184 random EMA prompts wherein tobacco use was reported in the past 15 min (*n =* 73 e-cigarette only (39.7% of episodes) vs. *n =* 111 combustible tobacco product (60.3% of episodes).

Table 2 presents results from the bivariate GEE models. Baseline FTCD scores, use of cartridge or tank e-cigarette, device, craving, negative affect, activities of eating drinking, or traveling, being in a public space (yes/no), and companionship (with tobacco user (yes/no)) were associated with the tobacco use episode type at *p* < 0.2. These variables were included in the multivariable regression model (Table 3). All the variables that reached significance in the bivariate GEE model primary analysis reached significance in the sensitivity analysis, with the addition of the activity of working/studying/reading (*p* < 0.2). 

The multivariable GEE model (Table 3) showed that high baseline cigarette dependence, as measured by the FTCD, was associated with lower odds of e-cigarette only vs. combustible tobacco product episodes (aOR 0.01, 95% CI (0.002–0.08); *p* < 0.001). Neither between- or within-subjects negative affect or craving scores were associated with e-cigarette only episodes. Activities of eating/drinking (aOR 0.20, 95% CI (0.08–0.49); *p* = 0.001) and being in the companionship of a person who smoked cigarettes (aOR 0.13, 95% CI (0.04–0.43); *p* = 0.001) were associated with lower odds of e-cigarette only vs. combustible tobacco product use episodes. However, traveling (aOR 12.02, 95% CI (3.77–38.26); *p* ≤ 0.001) and being in a public space (aOR 2.76, 95% CI (1.10–6.96); *p* = 0.03) were associated with higher odds of e-cigarette only than combustible tobacco product episodes. The sensitivity analysis had similar findings, however using a cartridge device was associated with lower odds of e-cigarette only than cigarette smoking only episodes. 

## 5. Discussion

In this pilot EMA study of a sample of young adults who used both e-cigarettes and combustible tobacco products, we identified unique contextual factors that were associated with episode type, such as being in a public place, traveling or eating/drinking. As expected, we also found strong associations between cigarette dependence and combustible tobacco product use. These preliminary findings need to be replicated in larger samples and can help inform the design of future large-scale studies that further examine high-risk contextual factors for e-cigarette and combustible tobacco product use among young adults.

Our data showed that there were higher odds of e-cigarette only episodes in public spaces, such as work, school, or restaurants, compared to combustible tobacco product episodes. Since cigarette smoking is prohibited in most public places, these data may suggest that e-cigarette use is occurring in places where cigarette smoking is prohibited. Additionally, traveling was also associated with higher odds of e-cigarette use episodes. If traveling occurs via public transportation wherein cigarette smoking is not permitted, e-cigarettes may be preferred by young adults who are dual users if use can remain discreet and unnoticeable. It is also plausible that young adults prefer to use e-cigarettes in their own vehicles as they have less odor than cigarettes. Studies of young adults demonstrate that the convenience, discreetness, and similarity of e-cigarettes to cigarettes serve as both motives for use and barriers to quitting vaping [20,21,22]. Although smoke-free air laws have been shown to reduce cigarette smoking rates [23], state laws prohibiting e-cigarette use indoors have not been shown to be associated with reductions in e-cigarette use among young adults [24], suggesting that other intervention strategies may be needed to reduce e-cigarette use in this population.

Conversely, we also found that the odds of e-cigarette only episodes vs. combustible tobacco product episodes were lower during eating/drinking (and higher with combustible tobacco product use episodes). This finding may be due to eating/drinking being less pleasurable or satisfying during e-cigarette vaping than cigarette smoking. Eating and drinking (alcohol specifically) have been correlated with cigarette smoking in EMA studies of both heavy [25] and intermittent smokers [26]; among young adults eating/drinking has been shown to be associated with light or non-daily smoking [27]. Future research is needed to understand these relationships during e-cigarette use episodes.

The odds of e-cigarette only episodes were lower than combustible tobacco product episodes when being around a cigarette smoker. This finding aligns with research demonstrating that exposure to cigarette smoking increases cigarette smoking urge through visual cues and the desire for a shared social experience [28,29,30]. Many young adults report smoking almost exclusively in social settings with other cigarette smokers [31,32]. Interestingly, being around someone who was vaping did not increase the odds of e-cigarette use, as compared to combustible tobacco product use. Previous research has shown that exposure to e-cigarette use increases urge for both cigarette smoking and e-cigarettes among people who use both products, therefore it is plausible that similar levels of urge for cigarettes and for e-cigarettes could contribute to the finding of no differences in the odds of vaping compared to smoking when exposed to e-cigarette visual cues [28,29,30]. Future studies are needed to explore these possibilities further.

Of note, we did not find significant associations between negative affect or craving and episode type, which may be due in part to the specific sample. Whereas many EMA studies of adults who are dual users include cigarette smokers who smoke more than five cigarettes/day [33], this study examined a young adult population exclusively; many of whom smoked less than five cigarettes per day and had low levels of cigarette dependence (defined as an FTCD score of ≤2). It is possible that individuals with this low level of dependence may not have used nicotine for affect regulation or the mitigation of craving. It is also possible that they reported on these measures after they had starting vaping/smoking, resulting in less variability in the craving and affect measures. Future studies are needed to further explore these relationships among similar populations as well as young adults with higher levels of nicotine dependence.

This study has several limitations. First and foremost, the small sample sizes limited the ability to detect all meaningful associations or model interactions. Further, the confidence levels of several estimates are very large, and can only be interpreted in an exploratory manner. We were unable to identify factors uniquely associated with dual use (as compared to e-cigarette or combustible tobacco product only use) due to these small sample sizes. However, we did perform a sensitivity analysis comparing e-cigarette only to cigarette only episodes, which suggested that the associations, with the addition of device type (cartridge), remained significant when dual use episodes were excluded. Future research is needed to understand whether device type has a unique role in episode type. Second, our findings do not generalize to larger, representative samples of young adults or other unique populations; we collected data in 2018 from a non-representative sample of young adults in CT. For example, national data suggests almost 75% of adults who use both e-cigarettes and cigarettes are non-Hispanic White [34], however the majority of our sample was non-Hispanic Black or Hispanic. Lastly, these 2018 data may not reflect patterns of dual use with newer e-cigarette devices.

## 6. Conclusions

In summary, this pilot EMA study adds to the small but growing literature differentiating factors associated with e-cigarette use and combustible tobacco product smoking among young adults. To reduce the overall burden of tobacco-related disease, an understanding of these dual use patterns is required to design tailored and dynamic cessation interventions that consider the unique triggers for e-cigarette or combustible tobacco product use. Future research with larger populations is needed to further identify real-time contextual factors that could be targets for potential interventions.

## Figures and Tables

**Table 1:** Baseline characteristics (*n* = 29).

	*n* (%) or Mean [SD]	
Women, *n* (%)	14	(48)
Age, Years [SD]	22.9	[3.4]
Race/Ethnicity, *n* (%)		
White Non-Hispanic	14	(48)
Black Non-Hispanic	3	(10)
Hispanic	10	(35)
Other Race/Ethnicity	2	(7)
Highest Level of Education, *n* (%)		
High School	18	(62)
Some College/College	11	(38)
Insurance, *n* (%)		
Public	17	(59)
From Parent	8	(28)
Private	3	(10)
None	1	(3)
Baseline FTCD Score, Mean [SD]	2.6	[2.4]
Past 28-day E-cigarette Use Behaviors		
Times/Day, Mean [SD]	4.9	[0.2–60]
Puffs/Day, Mean [SD]	3.4	[0.3–193]
# Refills/Day, Median [range]	0.07	[0–1]
E-cigarette Device Type, *n* (%)		
Cartridge	12	(41)
Tank	12	(41)
Mod	2	(7)
Other	3	(11)
Cigarettes/Day, *n* (%)		
Non-Daily	9	(31)
1 to 5	10	(35)
6 to 10	3	(10)
10 to 20	6	(21)
21+	1	(3)
Combustible Tobacco Product Used in Past Week, *n* (%)		
Cigarettes Only	21	(72)
Cigarette + Hookah	3	(10)
Cigarettes + Cigars	2	(7)
Cigarettes + Cigars + Hookah	3	(10)

Percentages may not total 100% due to rounding.

**Table 2:** Bivariate associations between episode type and baseline, subjective, and contextual factors (*n* = 29).

	Primary Analysis				Sensitivity Analysis			
	E-Cigarette Only vs. Combustible Tobacco Product Episodes (*n =* 184 Episodes)				E-Cigarette only vs. Cigarette only Episodes (*n =* 162 Episodes)			
	OR	LCI	UCI	*p*-Value	OR	LCI	UCI	*p*-Value
**Baseline Factors**								
Demographics								
Age	0.88	0.69	1.12	0.3	0.85	0.66	1.10	0.22
Sex (Female vs. Male)	0.42	0.10	1.747	0.2	0.42	0.09	1.96	0.3
Black/Other Race (yes vs. no)	0.92	0.23	3.75	0.9	0.87	0.19	3.88	0.9
Hispanic (yes vs. no)	1.28	0.30	5.48	0.7	1.38	0.30	6.39	0.7
1+ Years of College (yes/no)	0.80	0.21	2.99	0.7	0.78	0.19	3.26	0.7
Public/No Insurance (yes vs. no)	0.435	0.104	1.822	0.22	0.445	0.097	2.042	0.3
Baseline FTCD Score (3+ vs. ≤2)	0.04	0.01	0.15	<0.0001	0.03	0.01	0.15	<0.0001
Device Type (yes vs. no)								
Cartridge	0.28	0.06	1.26	0.09	0.21	0.04	1.01	0.05
Tank	3.94	0.89	17.46	0.07	5.36	0.99	28.97	0.05
Other Device	1.39	0.33	5.82	0.6	1.93	0.41	9.17	0.4
**Subjective Factors**								
Between-Subjects Craving	0.60	0.30	1.17	0.1	0.68	0.30	1.56	0.4
Within-Subject Craving	0.80	0.53	1.11	0.2	0.84	0.59	1.19	0.3
Between-Subject Negative Affect	0.91	0.83	1.00	0.06	0.92	0.83	1.03	0.17
Within-Subject Negative Affect	1.00	0.94	1.07	1.0	0.99	0.93	1.05	0.8
**Contextual Factors (yes vs. no)**								
Activity								
Eating/Drinking	0.17	0.06	0.52	0.002	0.15	0.05	0.47	0.001
Working/Reading/Studying	1.46	0.59	3.64	0.4	2.45	0.73	8.30	0.1
Traveling	2.65	0.92	7.65	0.07	6.47	0.85	49.34	0.1
Socializing	1.34	0.51	3.53	0.6	1.10	0.40	2.98	0.9
Location								
Public Space	1.80	0.96	3.38	0.06	1.82	0.97	3.43	0.1
Companionship								
With Someone Smoking Cigarettes	0.13	0.05	0.32	<0.0001	0.13	0.05	0.36	<0.0001
With Someone Vaping	0.80	0.27	2.38	0.7	1.24	0.33	4.62	0.7
With Non-Smoker	1.79	0.62	5.15	0.3	1.68	0.55	5.18	0.4
Alone *	3.80	1.70	8.51	0.001	2.24	3.89	1.61	0.003

Model adjusted for timing of the assessment (weekend, time of day (am vs. pm), and day of study (1 to 7)). OR = Odds Ratio; LCI = Lower 95% Confidence Interval; UCI = Upper 95% Confidence Interval. * Variable not included in multivariable model due to collinearity with variable “someone smoking cigarettes”.

**Table 3:** Multivariable associations between episode type and baseline, subjective, and contextual factors (*n* = 29 participants).

	Primary Analysis				Sensitivity Analysis			
	E-Cigarette Only vs. Combustible Tobacco Product Episodes (*n* = 184 Episodes)				E-Cigarette Only vs. Cigarette only Episodes (*n* = 162 Episodes)			
	aOR	LCI	UCI	*p*-Value	aOR	LCI	UCI	*p*-Value
**Baseline Factors**								
Baseline FTCD Score (3+ vs. ≤2)	0.01	0.002	0.08	<0.0001	0.01	<0.001	0.06	<0.0001
Device Type								
Cartridge (Yes vs. No)	0.27	0.03	2.56	0.3	0.06	0.01	0.40	0.004
Tank (Yes vs. No)	1.45	0.10	20.14	0.8	0.71	0.08	6.334	0.8
**Subjective Factors**								
Between-Subjects Craving	1.70	0.77	3.73	0.2	1.86	0.69	5.00	0.2
Within-Subject Craving	0.64	0.32	1.30	0.2	0.66	0.32	1.34	0.3
Between-Subject Negative Affect	0.99	0.86	1.15	0.9	1.03	0.91	1.18	0.6
Within-Subject Negative Affect	1.04	0.87	1.24	0.7	0.88	0.74	1.04	0.1
**Contextual Factors (yes vs. no)**								
Activity								
Eating/Drinking	0.20	0.08	0.49	0.001	0.17	0.03	0.97	0.046
Traveling	12.02	3.77	38.26	<0.0001	39.83	6.19	256.1	0.0001
Working/Reading/Studying	--	--	---	--	5.69	0.29	112.463	0.3
Location								
Public Spaces	2.57	1.01	6.58	0.048	2.29	0.54	9.71	0.3
Companionship								
With Smoker	0.13	0.04	0.43	0.001	0.08	0.02	0.36	0.001

Model adjusted for timing of the assessment (weekend, time of day (am vs. pm), and day of study (1 to 7)). The significance level was 0.05. aOR = adjusted Odds Ratio; LCI = Lower 95% Confidence Interval; UCI = Upper 95% Confidence Interval.

## Data Availability

Please contact authors for data requests.
